# A Bioinformatic Analysis of Correlations between Polymeric Immunoglobulin Receptor (PIGR) and Liver Fibrosis Progression

**DOI:** 10.1155/2021/5541780

**Published:** 2021-04-10

**Authors:** Yuan Zhang, Wenjun Lu, Xiaorong Chen, Yajuan Cao, Zongguo Yang

**Affiliations:** ^1^Department of Integrative Medicine, Shanghai Public Health Clinical Center, Fudan University, Shanghai 201508, China; ^2^Department of Rheumatology and Immunology, The People's Hospital of Danyang, Affiliated Danyang Hospital of Nantong University, Jiangsu 212300, China; ^3^Central Laboratory, Shanghai Pulmonary Hospital, School of Medicine, Tongji University School of Medicine, Shanghai 200433, China; ^4^Clinical Translation Research Center, Shanghai Pulmonary Hospital, Tongji University School of Medicine, Shanghai 200433, China

## Abstract

**Objective:**

This study is aimed at investigating the enriched functions of polymeric immunoglobulin receptor (PIGR) and its correlations with liver fibrosis stage.

**Methods:**

PIGR mRNA expression in normal liver, liver fibrosis, hepatic stellate cells (HSCs), and hepatitis virus infection samples was calculated in Gene Expression Omnibus (GEO) and Oncomine databases. Enrichment analysis of PIGR-related genes was conducted in Metascape and Gene Set Enrichment Analysis (GSEA). Logistic model and ROC curve were performed to evaluate the correlations between pIgR and liver fibrosis.

**Results:**

PIGR mRNA was upregulated in advanced liver fibrosis, cirrhosis compared to normal liver (all *p* < 0.05). PIGR mRNA was also overexpressed in activated HSCs compared to senescent HSCs, liver stem/progenitor cells, and reverted HSCs (all *p* < 0.05). Enrichment analysis revealed that PIGR-related genes involved in the defense response to virus and interferon (IFN) signaling. In GEO series, PIGR mRNA was also upregulated by hepatitis virus B, C, D, and E infection (all *p* < 0.05). After adjusting age and gender, multivariate logistic regression models revealed that high PIGR in the liver was a risk factor for liver fibrosis (OR = 82.2, *p* < 0.001). The area under curve (AUC), positive predictive value (PPV), negative predictive value (NPV), sensitivity, and specificity of PIGR for liver fibrosis stage >2 were 0.84, 0.86, 0.7, 0.61, and 0.90.

**Conclusion:**

PIGR was correlated with liver fibrosis and might involve in hepatitis virus infection and HSC transdifferentiation.

## 1. Introduction

The polymeric immunoglobulin receptor (PIGR) is exclusively originated from intestinal epithelial cells. It captured and transcytosed dimeric IgA (dIgA) from lamina propria to intestinal lumen across epithelial cells and participated in mucosal immune system [1, 2]. Previous evidences revealed that proinflammatory cytokines released by innate and adaptive immune cells including interferon, tumor necrosis factor (TNF), interleukins, and lymphotoxin could stimulate the expression of PIGR [1–9]. Except for exerting receptor function, the extracellular portion of PIGR is also responsible for intracellular neutralization of some viruses [10, 11].

Recently, PIGR has been proved to be involved in the human tumorigenesis and malignancies [9, 12–14]. As a vital inflammatory mediator [1], PIGR played an important role in hepatitis B (HBV) infection, chronic liver inflammation, tumor growth, recurrence, and metastatic progression in liver cancer and pancreatic ductal adenocarcinoma [9, 12, 14]. Mechanistically, PIGR had a cross talk of transforming growth factor-*β* with inflammatory mediators like tumor necrosis factor-*α*, interferon-*γ*, and interleukin-4, resulting in the induction of epithelial-mesenchymal transition (EMT) [13]. Moreover, PIGR upregulation increased the nuclear translocation of Smad2/3, leading to the activation of Smad pathway [12]. Rac1/CDC42-MEK/ERK cascade11 was also proved to be potential mechanism of PIGR-related cancer malignancy [14]. We previously found that PIGR involved in the activation of ribosome pathway and accounted for liver cancer recurrence [15]. Upregulated in alcoholic liver disease, PIGR isoform X1 has been identified as a predictor for liver fibrosis [16]. Additionally, PIGR was overexpressed in cirrhosis compared to nonalcoholic fatty liver disease. A global correlation map of clinical and proteomic data strongly associated PIGR with liver cirrhosis [17].

In this study, we aimed to investigate the expression of PIGR according to liver fibrosis, status of hepatic stellate cells (HSCs), and hepatitis virus infection. By identifying the associations between PIGR and liver fibrosis progression, we hope to offer novel insights into the mechanisms of liver fibrosis, even in hepatocarcinogenesis.

## 2. Materials and Methods

### 2.1. Gene Expression Omnibus (GEO)

GEO database [18, 19] was searched with heading terms including “liver fibrosis,” “cirrhosis,” “hepatitis,” “hepatitis virus,” “hepatitis A (HAV),” “hepatitis B,” “hepatitis C (HCV),” “hepatitis D (HDV),” and “hepatitis E (HEV).” All the series with expression profiling by array were included. No sample type and organism type restriction. Platforms and samples of GEO series were downloaded from https://www.ncbi.nlm.nih.gov/geo/.Raw.CEL files of the microarray from each GEO dataset were normalized by quantile method of Robust Multichip Analysis (RMA) from R affy package [20]. Gene expression comparison was calculated by Limma package in R program version 4.0 [21]. The details of GEO series included in this analysis were summarized as Supplementary Table S1.

### 2.2. Oncomine

The Oncomine is a cancer microarray database and web-based integrated data mining platform aimed at facilitating discovery from genome-wide expression analyses [22]. With more than 700 independent datasets and a collection of over 18000 microarray experiments, the Oncomine platform provides solutions that can compute gene expression signatures, clusters, and gene-set modules, automatically extracting biological insights from the data [22, 23]. Studies compared PIGR between normal liver and cirrhosis samples were selected without threshold, fold change, and gene rank restriction in Oncomine database (https://www.oncomine.org/).

### 2.3. The Human Protein Atlas (HPA)

The HPA program mapped all the human proteins in cells, tissues, and organs using an integration of various omics technologies, including antibody-based imaging, mass spectrometry-based proteomics, transcriptomics, and systems biology [24]. PIGR protein in tissue atlas and PIGR mRNA in cell type atlas of liver were obtained from HPA database.

### 2.4. DisGeNET

DisGeNET is a discovery platform containing one of the largest publicly available collections of genes associated to human diseases [25–27]. Associated diseases of PIGR were searched in DisGeNET version 6.0 (https://www.disgenet.org/). Summaries and evidences of gene-disease associations (GDA) including semantic type, scores of GDA, and numbers of publications in PubMed were all obtained.

### 2.5. Enrichment Analysis

Using Similar Genes Detection function in Gene Expression Profiling Interactive Analysis (GEPIA) database, top 100 similar genes of PIGR with similar expression pattern in LIHC normal liver dataset were identified [28]. Protein-Protein Interaction analysis (PPI) for PIGR was investigated by STRING version 11.0 (https://string-db.org/) and STITCH version 5.0 (http://stitch.embl.de/) databases. All these interacted genes and similar genes of PIGR in GEPIA, STRING, and STITCH were included in Metascape for enrichment analysis [29]. Top ten Kyoto Encyclopedia of Genes and Genomes (KEGG) pathway, Gene ontology (GO) biological process, and Reactome enrichment analysis were also investigated in Molecular Signatures Database in Gene Set Enrichment Analysis (GSEA, http://software.broadinstitute.org/gsea/index.jsp) version 4.1 with a false discovery rate (FDR) *p* value < 0.05 [30, 31].

### 2.6. Statistical Analysis

Differences of PIGR expression levels between the individual groups were analyzed using Student's *t* test or Mann–Whitney test based on variable types by GraphPad Prism 8 (GraphPad Software, San Diego, CA, USA). Parameters associated with the liver fibrosis stage were assessed by univariate and multivariate logistic regression by Stata software version 16.0 (Stata Corp LLC, Texas, USA). Results were reported as odds ratios (OR) with 95% confidence intervals (CI). OptimalCutpoints package [32] in R program was used to perform ROC analysis to evaluate predictive values of potential factors for the liver fibrosis stage. A two-tailed *p* < 0.05 were considered significant for all tests.

## 3. Results

### 3.1. PIGR Expression in Different Stages of Liver Pathology

**Figure 1:**
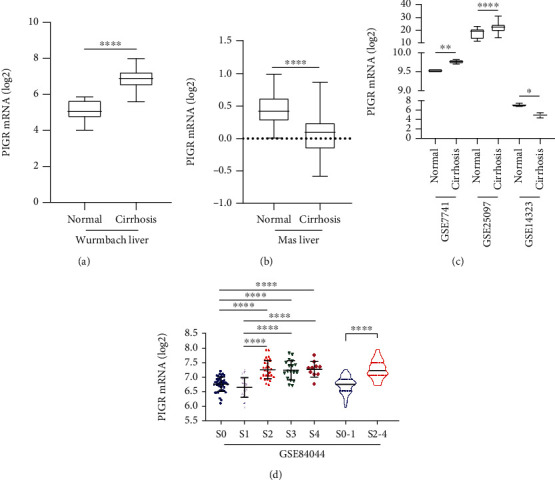
PIGR mRNA expression between normal and cirrhosis samples. PIGR was upregulated in cirrhosis than that in normal samples in Wurmbach Liver (*p* < 0.0001, a) and downregulated in cirrhosis in Mas Liver (*p* < 0.0001, b). In GEO series, PIGR was overexpressed in cirrhosis samples compared to normal livers in GSE7741 and GSE25097 and downregulated in cirrhotic samples in GSE14323 (c). Compared to liver fibrosis stages 0-1, PIGR elevated in patients with liver fibrosis stages 2-4 (*p* < 0.0001, d).

In Oncomine database, Wurmbach Liver and Mas Liver datasets reported PIGR comparison between cirrhosis and normal liver. Microarray experiments of 13 cirrhosis samples and 10 normal liver samples in Wurmbach Liver dataset were addressed in Human Genome U133 Plus 2.0 Array platform [33], and Transcriptome levels of PIGR in 19 normal liver samples and 58 cirrhosis samples in Mas Liver dataset were examined in Human Genome U133A 2.0 Array platform [34]. As shown in Figure 1(a), PIGR mRNA was significantly upregulated in cirrhosis tissues than that in normal liver in Wurmbach Liver dataset (*p* < 0.0001, Figure 1(a)). Conversely, PIGR mRNA was downregulated in cirrhosis samples than that in normal liver in Mas Liver dataset (*p* < 0.0001, Figure 1(b)). GEO series were also selected for investigating PIGR expression between cirrhotic and normal livers. The details of the included GEO series were summarized in Supplementary Table S1. As shown in Figure 1(c), PIGR mRNA was significantly overexpressed in cirrhotic samples than that in normal livers in GSE7741 and GSE25097 (*p* < 0.01 and *p* < 0.0001, respectively, Figure 1(c)). On the contrary, PIGR was downregulated in cirrhosis compared to that in healthy individuals in GSE14323 (*p* < 0.05, Figure 1(c)). In GSE84044, liver samples of 124 chronic hepatitis B (CHB) patients were examined [35]. As shown in Figure 1(d), PIGR mRNA was significantly overexpressed in CHB patients with fibrosis stage ≥2 compared to that in patients with fibrosis stage <2 (all *p* < 0.001, Figure 1(d)).

### 3.2. PIGR Expression in Liver Cells and HSCs

**Figure 2:**
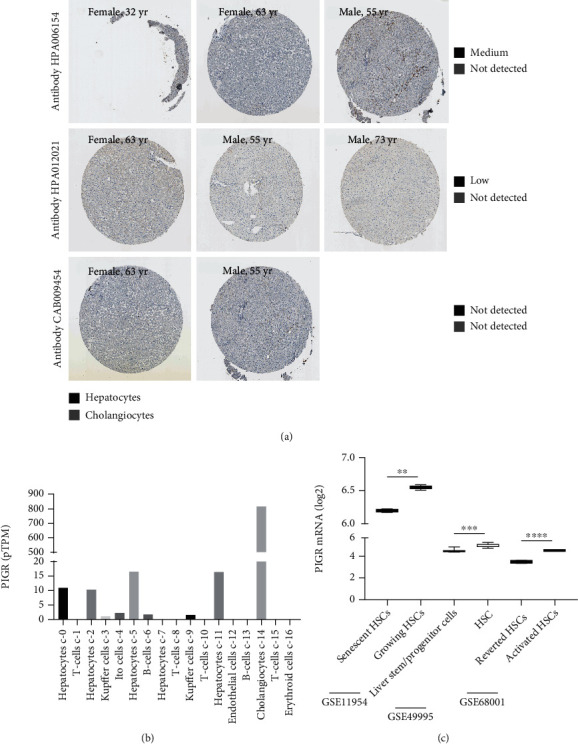
PIGR protein staining in liver tissues (a); PIGR mRNA expression in the single cell type clusters identified in liver tissue (b); and PIGR mRNA expression comparison in different status HSCs (c).

PIGR protein in liver tissues was obtained from HPA database. As shown in Figure 2(a), PIGR was not detected in all eight cholangiocytes, while it was medium/low staining in six of eight hepatocytes (Figure 2(a)). PIGR RNA expression in the single cell type clusters identified in liver tissue was summarized in Figure 2(b). PIGR mainly expressed in cholangiocytes, followed by hepatocytes (Figure 2(b)).

Considered the pivotal roles of HSCs in the development of liver fibrosis, PIGR mRNA expression levels were identified in HSCs in GEO series including GSE11954 [36], GSE49995 [37], and GSE68001 [38]. In GSE11954, two separate preparation of activated HSCs were treated with DNA damaging agent to induce senescence or vehicle to remain growing [36]. Compared to senescent HSCs, PIGR in growing HSCs was significantly overexpressed (*p* < 0.01, Figure 2(c)). In GSE49995, PIGR expression in 7 samples of adult-derived human liver stem/progenitor cells (ADHLSCs) and 7 samples of HSCs were measured [37]. PIGR mRNA was significantly upregulated in HSCs compared to that in ADHLSCs (*p* < 0.001, Figure 2(c)). In GSE68001, HSCs were isolated from healthy liver and culture-activated as aHSCs, and aHSCs were reverted by reverting medium and displayed as a quiescent-like phenotype (rHSCs) [38]. Compared to rHSCs, PIGR was significantly upregulated in aHSCs (*p* < 0.001, Figure 2(c)).

### 3.3. Enrichment of Similar/Interactive Genes of pIgR

**Figure 3:**
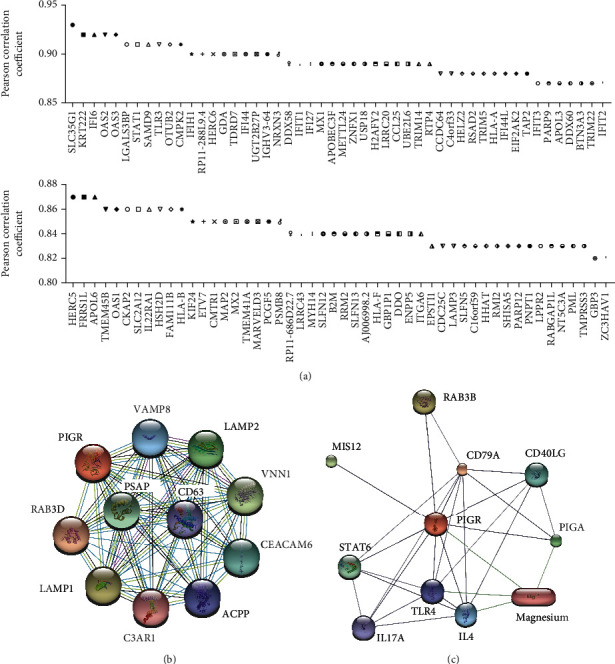
Top 100 similar genes of PIGR in GEPIA database (a) and protein-protein interaction (PPI) with PIGR in STRING (b) and STITCH (c).

**Figure 4:**
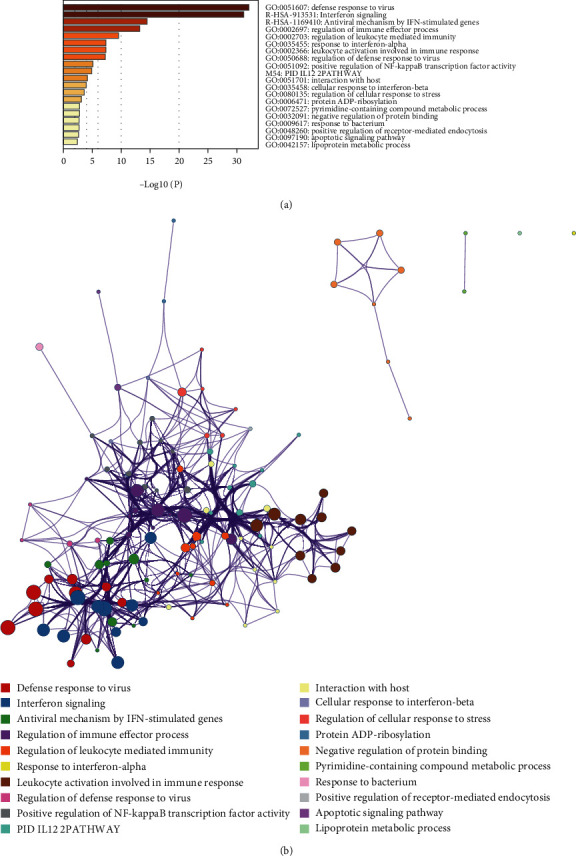
Enrichment of similar/interacted genes of PIGR in Metascape database.

**Figure 5:**
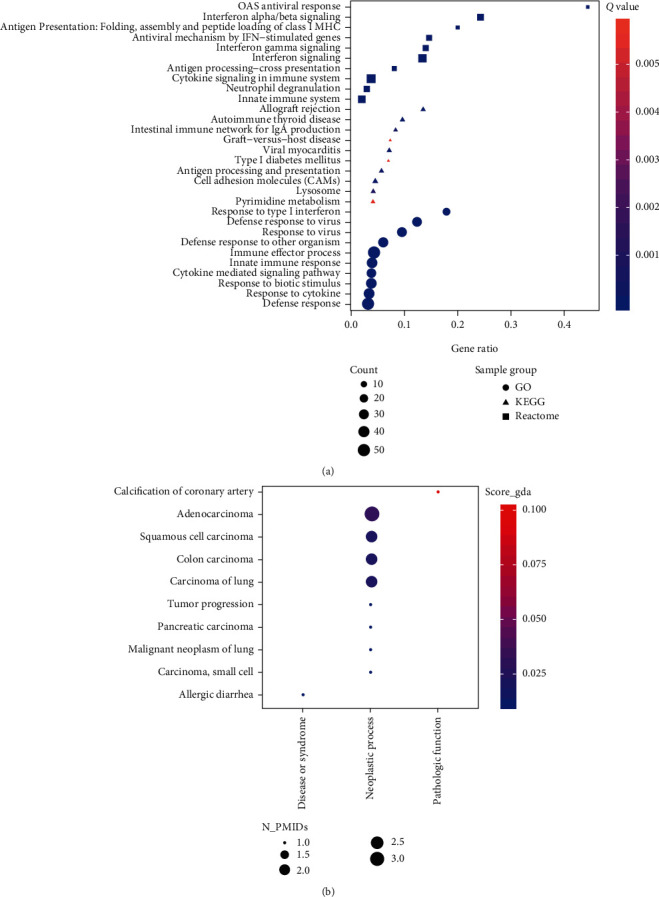
Enrichment of similar/interacted genes of PIGR in GSEA (a) and PIGR-related diseases in DisGeNET (b).

Top 100 similar genes of PIGR in LIHC normal liver dataset were identified in GEPIA database (Figure 3(a)). PPI of PIGR was evaluated in STRING and STITCH databases (Figures 3(b) and 3(c), respectively). Enrichment analysis of similar/interacted genes of PIGR was calculated in Metascape. As summarized in Figure 4, defense response to virus, interferon (IFN) signaling, and regulation of immune effector process were mainly enriched (Figures 4(a) and 4(b)). Additionally, KEGG, Reactome, and GO enrichment of similar/interacted genes of PIGR were reevaluated in GSEA. In line with results in Metascape, defense response to virus, response to virus, and response to type I IFN were mainly enriched in GO in GSEA. Antiviral response and IFN alpha/beta signaling were mainly enriched in Reactome. Moreover, pathways involved in immune responses were enriched in KEGG in GSEA (Figure 5(a)).

In DisGeNET, neoplastic process including adenocarcinoma, squamous cell carcinoma, colon carcinoma, carcinoma of lung, tumor progression, pancreatic carcinoma, malignant neoplasm of lung, and small cell carcinoma was mainly associated with PIGR based on the current evidences from publications in PubMed (Figure 5(b)). As shown in Figure 5(b), no publications have investigated the correlations between PIGR and liver fibrosis.

### 3.4. PIGR Expression in Hepatitis Virus Infection

**Figure 6:**
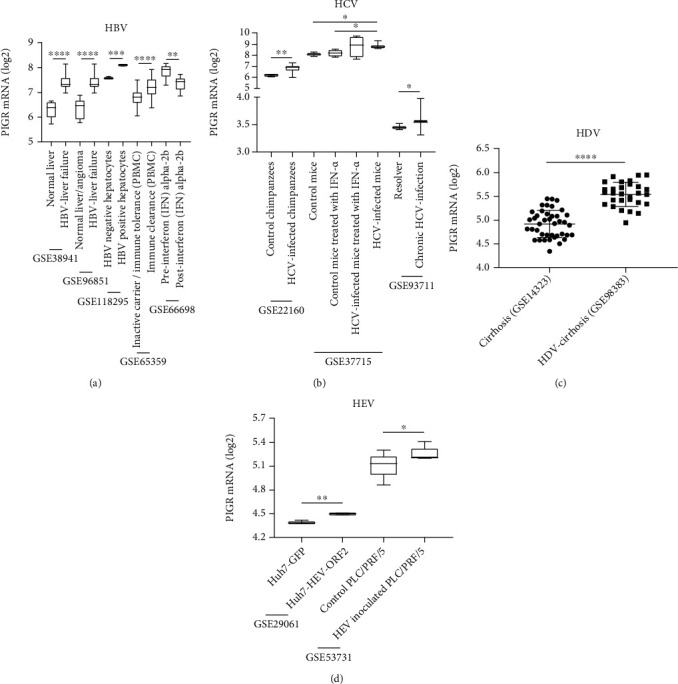
PIGR mRNA expression levels according to hepatitis virus B (a), C (b), D (c), and E (d) infection.

According to the enrichment analysis results, PIGR was mainly involved in virus- and IFN-related responses. Hence, we identified the PIGR expression levels in different hepatitis virus infections. In HBV infection, PIGR mRNA was significantly upregulated in HBV-associated liver failure samples compared to normal individuals and liver angioma in GSE38941 [39] and GSE96851 [40] (both *p* < 0.001, Figure 6(a)). Compared to HBV-negative samples, PIGR mRNA was significantly overexpressed in HBV-positive liver samples in GSE118295 (*p* < 0.01, Figure 6(a)). In addition, PIGR mRNA was also significantly upregulated in PBMC samples from immune clearance CHB patients compared to that from inactive carrier and immune tolerance ones (*p* < 0.001, Figure 6(a)). Intriguingly, PIGR mRNA was significantly higher in pre-IFN alpha-2b livers than that in post-IFN alpha-2b samples (*p* < 0.01, Figure 6(a)) [41].

Compared to control chimpanzees, PIGR mRNA was significantly overexpressed in HCV-infected ones in GSE22160 [42] (*p* < 0.01, Figure 6(b)). Compared to control mice and control mice treated with IFN-alpha, PIGR mRNA was significantly upregulated in HCV-infected mice in GSE37715 (both *p* < 0.05, Figure 6(b)). In addition, PIGR mRNA was significantly higher in chronic HCV-infection than that in resolver in GSE93711 [43] (*p* < 0.05, Figure 6(b)).

PIGR mRNA was significantly upregulated in HDV-related cirrhosis in GSE98383 [44] compared to cirrhosis samples from GSE14323 [34] (*p* < 0.001, Figure 6(c)).

PIGR mRNA was also significantly overexpressed in Huh7 cells with recombinant adenovirus encoding the HEV ORF2 compared to that in adenovirus encoding the green fluorescent protein in GSE29061 [45] (*p* < 0.01, Figure 6(d)). In addition, PIGR mRNA was significantly upregulated in PLC/PRF/5 cells inoculated with HEV than that in PLC/PRF/5 cells inoculated with serum-free DMEM/199 medium in GSE53731 [46] (*p* < 0.05, Figure 6(d)).

### 3.5. Associations between pIgR and Fibrosis Stage in CHB Patients

**Figure 7:**
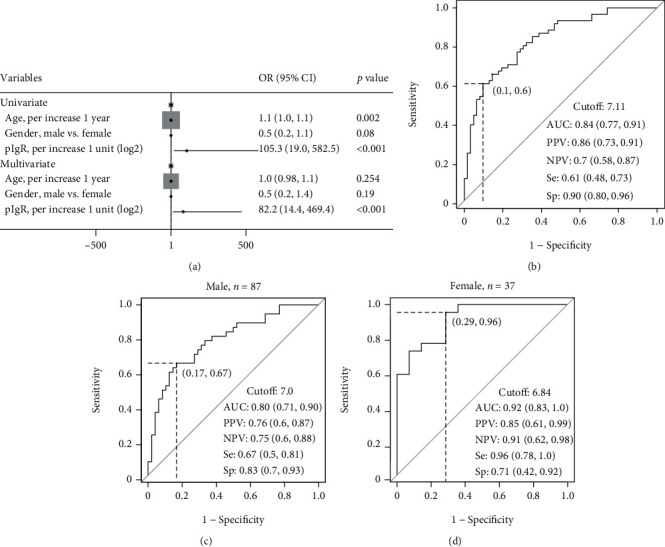
Logistic regression analysis of parameters associated with liver fibrosis stage ≥2 in chronic hepatitis B patients (a) and ROC curves of PIGR for liver fibrosis stage ≥2 in CHB patients (b–d) in GSE84044.

In GSE84044 [35], information including liver fibrosis stage, age, and gender of 124 CHB patients were obtained from GEO database. After adjusting age and gender, multivariate logistic regression models revealed that high PIGR in liver was a risk factor for liver fibrosis (OR = 82.2, 95%CI = 14.4–469.4, *p* < 0.001, Figure 7(a)). The optimal cutoff of PIGR for predicting liver fibrosis stage ≥2 was 7.11. The area under curve (AUC), positive predictive value (PPV), negative predictive value (NPV), sensitivity, and specificity were 0.84, 0.86, 0.70, 0.61, and 0.90, respectively (Figure 7(b)).

We conducted subgroup analysis by gender. In male population, the optimal cutoff of PIGR for predicting liver fibrosis stage ≥2 was 7.0. The AUC, PPV, NPV, sensitivity, and specificity were 0.80, 0.76, 0.75, 0.67, and 0.83, respectively (Figure 7(c)). In female, the optimal cutoff of PIGR for predicting liver fibrosis stage ≥2 was 6.84. The AUC, PPV, NPV, sensitivity, and specificity were 0.92, 0.85, 0.91, 0.96, and 0.71, respectively (Figure 7(d)).

## 4. Discussion

PIGR was generally known as a mediator of transcytosis of polymeric immunoglobulins, accelerating the secretion of IgA and IgM and comprising the defense line against infection [1]. Recently, Nallagangula et al. and Niu et al. revealed the associations between PIGR and liver cirrhosis [16, 17]. In addition, Ai et al., Yue et al., and our previous studies have uncovered the unrecognized roles of PIGR in the promotion of tumorigenesis and metastases [12, 14, 15, 47]. On the other side, they identified the potential inflammation links between HBV infection and cancer malignancy [12, 14]. Considering the underlying correlations between hepatitis virus infection, liver fibrosis, and hepatocarcinogenesis, we assumed that PIGR might participated in the liver fibrosis progression, which resulted in liver malignancies.

In this analysis, we found that PIGR was apparently upregulated in advanced liver fibrosis. And high PIGR expression could reliably predict the liver fibrosis stage. Our previous publication revealed that PIGR was overexpressed in tumor tissues compared to adjacent tissues in hepatocellular carcinoma [47]. Thus, PIGR might promote the processes of liver fibrosis, cirrhosis, and hepatocarcinogenesis in the chronic liver diseases. Multiple intracellular signaling pathways including Janus kinase-signal transduction and activator of transcription (JAK-STAT), NF-*κ*B, and mitogen-activated protein kinase (MAPK) are involved in the regulation of PIGR [1–3]. Abnormal cytokine cross talk between epithelium and inflammatory cells contributed to fibrosis [48]. And the JAK-STAT, NF-*κ*B, and MAPK pathways were the main signaling in regulating liver fibrosis and regeneration induced by these cytokines [49–51]. Given the recently established connections between PIGR, proinflammatory cytokines, and signaling pathways including JAK-STAT, NF-*κ*B, and MAPK, our findings partly raise the underlying possibility that PIGR may facilitate liver fibrosis via proinflammatory cytokine-induced pathways.

HSCs and hepatitis virus infection are vital factors in liver fibrosis progression. Our results revealed that PIGR expression is in line with the activation of HSCs and the status of hepatitis virus infection. HSCs play a key role in the initiation, progression, and regression of liver fibrosis [52, 53], and activation of HSCs is the major cellular driver of liver fibrogenesis [54]. Proinflammatory cytokines, which could simulate the expression of PIGR, could promote transdifferentiation of quiescent HSCs into activated HSCs in fibrogenic liver and facilitate secretion of extracellular matrix molecules [52, 54]. On the other side, chronic hepatitis virus infection is one of the major risk factors for fibrotic liver diseases. Existed evidence showed that HBV e antigen, core antigen, and X proteins could directly induce activation and proliferation of HSCs [54, 55]. And HCV viral core and nonstructural proteins directly induce inflammatory and profibrogenic pathways including Ras/ERK, PI3K/AKT, and TGF-*β* signaling in HSCs [56]. Clinical studies also indicated that serum IgA level is positively correlated with the severity of liver fibrosis and functions as in independent predictor for cirrhosis [57, 58]. Thus, we drew the hypothesis that PIGR might be involved in the fibrosis progression via hepatitis virus- and HSC-related mechanisms.

This analysis has some limitations. Firstly, we conducted a bioinformatic analysis, without experimental validation of our results. Secondly, limited parameters were included in the logistic model; confounding variables might exist. Thirdly, PIGR expression between normal and cirrhotic samples is still controversial in multiple public datasets. Fourthly, PIGR was mainly detected in cholangiocytes and hepatocytes, not in HSCs, in HPA database. Hence, the causality between PIGR and liver fibrosis needs further confirmation, even though our results provided the potential correlations between PIGR, HSCs, hepatitis virus infection, and liver fibrosis progression, which might be helpful for identification of novel therapeutic targets of regression of liver fibrosis.

## Data Availability

Datasets of the current study are available from the NCBI Gene Expression Omnibus (https://www.ncbi.nlm.nih.gov/geo/), Oncomine (https://www.oncomine.org/), and the Human Protein Atlas (HPA, https://www.proteinatlas.org/) databases. All the datasets were available from the corresponding authors with reasonable request.

## Supplementary Materials

Supplementary Materials Supplementary Table S1: the details of GEO series included in this analysis.
