# Supplementary material for: A Bioinformatic Analysis of Correlations between Polymeric Immunoglobulin Receptor (PIGR) and Liver Fibrosis Progression
Source: Biomed Res Int. 2021 Apr 10;2021:5541780. doi: 10.1155/2021/5541780 (PMC8055406; doi:10.1155/2021/5541780)
Supplement: Supplementary Materials — Supplementary Table S1: the details of GEO series included in this analysis. [file 5541780.f1.docx]

**A bioinformatic analysis of correlations between polymeric immunoglobulin receptor (PIGR) and liver fibrosis progression**

**Supplementary Materials**

**Table S1.** The details of GEO series included in this analysis.

| Series | Contributors | Samples | Country | Platforms |
| --- | --- | --- | --- | --- |
| GSE7741 | Sarfraz S, et al, 2007 | 3 | Saudi Arabia | Affymetrix Human Genome U133 Plus 2.0 Array |
| GSE11954 | Krizhanovsky V, et al, 2008 | 4 | USA | Affymetrix Human Genome U133 Plus 2.0 Array |
| GSE14323 | Mas VR, et al, 2009 | 124 | USA | Affymetrix Human Genome U133A Array / Affymetrix Human Genome U133A 2.0 Array |
| GSE22160 | Yu C, et al, 2010 | 44 | USA | Affymetrix Human Genome U133 Plus 2.0 Array |
| GSE25097 | Zhang C, 2010 | 46 | USA | Rosetta/Merck Human RSTA Affymetrix 1.0 microarray, Custom CDF |
| GSE29061 | John L, et al, 2011 | 6 | Germany | Affymetrix Human Genome U133 Plus 2.0 Array |
| GSE37715 | Chayama K, 2012 | 15 | Japan | Affymetrix Human Genome U133 Plus 2.0 Array |
| GSE38941 | Nissim O, et al, 2012 | 27 | USA | Affymetrix Human Genome U133 Plus 2.0 Array |
| GSE49995 | Berardis S, et al, 2013 | 14 | Spain | Affymetrix Human Genome U219 Array |
| GSE53731 | Zhang F, 2013 | 10 | China | Affymetrix Human Genome U133 Plus 2.0 Array |
| GSE65359 | Lu M, et al, 2015 | 83 | China | Affymetrix Human Genome U133 Plus 2.0 Array |
| GSE66698 | Hsiao T, et al, 2015 | 17 | Taiwan, China | Affymetrix Human Genome U133 Plus 2.0 Array |
| GSE68001 | El Taghdouini A, et al, 2015 | 9 | Belgium | Affymetrix Human Genome U219 Array |
| GSE84044 | Wang M, et al, 2016 | 124 | China | Affymetrix Human Genome U133 Plus 2.0 Array |
| GSE93711 | Lauer GM and Wolski D, 2017 | 10 | USA | Affymetrix Human Genome U133A Array |
| GSE96851 | Chen Z, et al, 2017 | 34 | Italy | Affymetrix Human Genome U133 Plus 2.0 Array |
| GSE98383 | Diaz G, et al, 2017 | 74 | Italy | Affymetrix Human Genome U133 Plus 2.0 Array |
| GSE118295 | Xia Y, et al, 2018 | 6 | USA | Affymetrix Human Genome U133 Plus 2.0 Array |
